# Interpretable machine learning framework to predict gout associated with dietary fiber and triglyceride-glucose index

**DOI:** 10.1186/s12986-024-00802-2

**Published:** 2024-05-14

**Authors:** Shunshun Cao, Yangyang Hu

**Affiliations:** 1 Pediatric Endocrinology, Genetics and Metabolism, The Second Affiliated Hospital, Yuying Children’s Hospital of Wenzhou Medical University, Wenzhou, 325000 Zhejiang China; 2 Reproductive Medicine Center, Obstetrics and Gynecology, The Second Affiliated Hospital, Yuying Children’s Hospital of Wenzhou Medical University, Wenzhou, 325000 Zhejiang China

**Keywords:** Gout, Dietary fiber, Triglyceride-glucose index, NHANES, Machine learning

## Abstract

**Background:**

Gout prediction is essential for the development of individualized prevention and treatment plans. Our objective was to develop an efficient and interpretable machine learning (ML) model using the SHapley Additive exPlanation (SHAP) to link dietary fiber and triglyceride-glucose (TyG) index to predict gout.

**Methods:**

Using datasets from the National Health and Nutrition Examination Survey (NHANES) (2005–2018) population to study dietary fiber, the TyG index was used to predict gout. After evaluating the performance of six ML models and selecting the Light Gradient Boosting Machine (LGBM) as the optimal algorithm, we interpret the LGBM model for predicting gout using SHAP and reveal the decision-making process of the model.

**Results:**

An initial survey of 70,190 participants was conducted, and after a gradual exclusion process, 12,645 cases were finally included in the study. Selection of the best performing LGBM model for prediction of gout associated with dietary fiber and TyG index (Area under the ROC curve (AUC): 0.823, 95% confidence interval (CI): 0.798–0.848, Accuracy: 95.3%, Brier score: 0.077). The feature importance of SHAP values indicated that age was the most important feature affecting the model output, followed by uric acid (UA). The SHAP values showed that lower dietary fiber values had a more pronounced effect on the positive prediction of the model, while higher values of the TyG index had a more pronounced effect on the positive prediction of the model.

**Conclusion:**

The interpretable LGBM model associated with dietary fiber and TyG index showed high accuracy, efficiency, and robustness in predicting gout. Increasing dietary fiber intake and lowering the TyG index are beneficial in reducing the potential risk of gout.

**Supplementary Information:**

The online version contains supplementary material available at 10.1186/s12986-024-00802-2.

## Introduction

Gout is one of the most common forms of inflammatory arthritis caused by a sustained elevation of serum uric acid (UA) above the dissolved saturation level of 408 µmol/L, leading to an inflammatory response and pain due to the precipitation of sodium urate crystals and their deposition in the joints and soft tissues [1]. Epidemiologic surveys have shown that the prevalence of gout is 2.7–6.7% in countries with a Western lifestyle and that the heritability of hyperuricemia and gout ranges from 27 to 41% worldwide to about 30% in Europe [2]. While gout was once thought to be simply a disorder of purine metabolism, current research favors a multifactorial autoinflammatory disease, with only about 25% of simple hyperuricemia developing into gout [3]. Gout is associated with several important comorbidities, including chronic kidney disease, kidney stones, obesity, diabetes, and cardiovascular disease, and with the overall mortality rate of gout sufferers increasing each year, gout has become a public healthcare issue of concern worldwide [4–6].

Studies have shown that insulin resistance (IR) is prevalent in type 2 diabetes mellitus and obese populations, and the classic method for assessing insulin sensitivity is the hyperinsulinemic-euglycemic clamp test [7]. However, the complexity of clinical manipulation limits its use, and the triglyceride-glucose (TyG) index has been recognized as a novel and reliable indicator of IR [8]. A variety of dietary components such as dietary fiber, energy, and nutrients can influence the inflammatory response in the body, which has led researchers to focus on the relationship between diet and inflammation, and studies have shown that increasing dietary fiber intake can improve the inflammatory response in the body [9]. Studies have shown that urate crystals and inflammatory cell recruitment are strongly associated with gouty arthritis [10]. Vieira AT et al. showed that high dietary fiber promotes the reduction of the inflammatory response caused by urate crystals in mice, suggesting that diet plays a decisive role in the ability to modulate the inflammatory response [11]. However, studies on the correlation between the TyG index, dietary fiber, and gout have not been clarified, and it is innovative for us to link the TyG index, dietary fiber, and gout using a machine learning (ML) approach.

Data in the field of nutrition and metabolism are becoming complex and high-dimensional, and while traditional analytical approaches often rely on supervisory variables to study correlations with gout and do not fully capture the complexity of interactions between nutrition, metabolism, and disease, ML offers us the possibility of identifying the interactions of high-dimensional data variables [12, 13]. ML is a branch of Artificial Intelligence (AI) dedicated to performing complex tasks and analyzing data, effectively identifying complex relationships between variables that interact with each other, and dealing with unstructured data types that cannot be handled by traditional statistical techniques [14]. Previous ML models have not been widely used because they lacked interpretability and were difficult to be trusted by users [15]. Interpretable ML allows users to understand the model’s decision-making process and predicted outcomes, it emphasizes transparency and comprehension of the model and adds control and interpretation of algorithmic decisions that can identify potential problems in the model and avoid unnecessary errors and misinterpretations [16]. The purpose of this study was to develop six interpretable ML models using the National Health and Nutrition Examination Survey (NHANES) dataset and select the best model for predicting gout. We evaluated the best-performing model and used the interpretable ML model based on SHapley Additive exPlanations (SHAP) to assess the contribution of the TyG index and dietary fiber in predicting gout to develop individualized interventions for potential gout risk.

## Materials and methods

### Data source and study design

This was a cross-sectional study, and all data for this study were obtained from the Centers for Disease Control and Prevention NHANES dataset for 7 cycles from 2005 to 2018. Demographic, lifestyle, anthropometric, laboratory analysis, questionnaire interview, and dietary data from each NHANES cycle were merged according to SEQN (participant ID) using the Bernard D et al. method to generate a participant dataset containing all study variables [17]. The NHANES study was approved by the National Center for Health Statistics (NCHS) Ethics Review Board under approval no. # 2005- 06, # 2011– 17 [18]. Informed consent was obtained from all participants for this study.

**Fig. 1:**
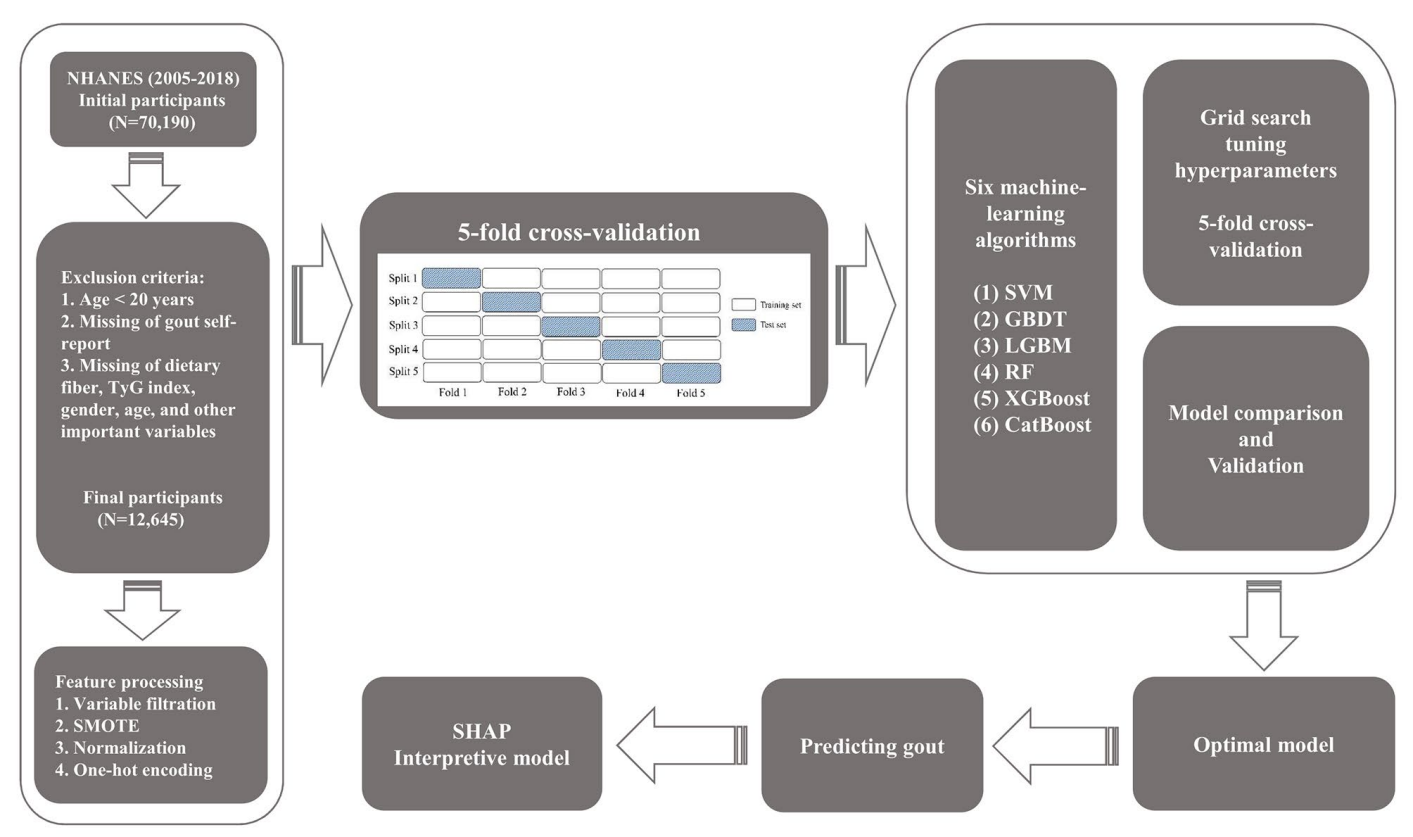
Flowchart for subject screening and study design

A total of 70,190 participants were initially assessed from 2005 to 2018, and Fig. 1 shows the study design and inclusion criteria, as well as participants excluded due to missing information on variables. Participants under the age of 20 were excluded from the dataset because many of the laboratory variables were not collected in this age group. Inclusion criteria included (1) participants aged ≥ 20 years; (2) participation in a dietary interview, fasting blood glucose, and triglyceride testing; and (3) complete participant self-reported gout information. Exclusion criteria included (1) participants aged < 20 years; (2) missing self-report of gout information; and (3) missing information on age, gender, fasting blood glucose, triglyceride, dietary interview, and other important variables. The final number of participants included in the study was 12,645.

### Identification and screening of features

Based on previous studies reported [19–21] and expert opinions (four independent specialists in nutrition, endocrinology, and metabolic diseases at the Second Affiliated Hospital of Wenzhou Medical University), we included demographics, laboratory parameters, lifestyle, anthropometric measurements, medical history, and dietary parameters as initial model features of the study containing 35 variables (Supplementary material 1). To eliminate redundant variables and multicollinearity in the data, to improve the generalization of the model, and to avoid overfitting, we used the caret R package to perform near-zero variance and correlation tests on the variables [22]. The height, weight, vitamin E (VitE), TyG-body mass index (BMI) index, waist circumference (WC), and homeostasis model assessment-IR (HOMA-IR) were excluded based on variable variance approximating zero and strong correlations, and biological features of participants were maximally preserved. We removed features with more than 20% missing values (apolipoprotein B and high-sensitivity C-reactive protein) in the cycle data. After processing, the selected dataset contained 12,645 participants, 27 variables, and limited missing data.

### Calculation of features

According to Belladelli F et al. [23], the TyG index is calculated as ln [triglycerides (mg/dL) × fasting glucose (mg/dL)/2]. Huang X et al. concluded that the TyG- BMI has the same novel index of IR as the TyG index, which is a better indicator of IR than the HOMA-IR, and its calculation formula is TyG-BMI = TyG index × BMI [24]. HOMA-IR was calculated as fasting blood glucose (mmol/L) × fasting insulin (µU/mL)/22.5 [25]. Systemic immune-inflammatory index (SII) was calculated as platelet count multiplied by neutrophil count divided by lymphocyte count as described by Mahemuti N et al. [26]. Dietary data were obtained from two dietary recall interviews for six dietary antioxidants including total vitamin A (VitA), total vitamin C (VitC), total vitamin E (VitE), total zinc, total selenium (Se), and total lutein + zeaxanthin (LZ). According to the method described by Wu M, the composite dietary antioxidant index (CDAI) was calculated by subtracting the mean value for each of the six antioxidants, dividing by the standard deviation to normalize it, and then summing it to obtain it [27].

### Definition of the label

We used self-reported gout as the label for the prediction model, and the 2005–2018 NHANES data initially included in the study were for all-age participants, with the age limit for participation in the gout self-report interview questionnaire being 20 years of age and older. Therefore, we excluded patients under 20 years of age. We confirmed whether participants had gout by self-reported data from the MCQ160N on the interview questionnaire. When study participants were asked, “Has a doctor ever told you that you have gout?” Confirmation code 1 indicated gout, confirmation code 2 indicated no gout, confirmation code 7 indicated refusal to answer, and confirmation code 9 indicated not sure.

### Handling missing values

Missing data is a real problem that is often encountered in clinical medical studies. Simple direct deletion of data will result in the loss of valuable information and waste of resources, so the filling of missing data is a reasonable and realistic operation. Conventional data-filling methods such as multiple interpolation and median interpolation are unable to deal with data interactions and nonlinearities of variables in the face of high dimensional and large sample data, so algorithm-based data-filling can more accurately maintain the overall characteristics of the data [28]. We used the missForest R package (hyperparameters: maxiter = 10, ntree = 1000, verbose = TRUE) to interpolate variables with a few missing data. The method is based on using the missing data problem as a model prediction, and each variable in turn is predicted using a fitted Random Forest (RF) regression model to predict the missing data for the dependent variable, and the study confirms that this method outperforms non-algorithm-based interpolation methods [29, 30].

### Model development strategy

To better reflect the performance of the model on unknown data, we use nested 5-fold cross-validation [31]. It exists inner and outer loops for a total of 5 iterations, and for each iteration in the 5-fold cross-validation, there exists a nested 5-fold cross-validation. An inner loop is a 5-fold cross-validation with a grid search for the best hyperparameters of the model to provide the outer loop with the best hyperparameters of the model. The outer loop is to provide the inner loop with 4 subsets of data for training the model while retaining 1 subset of data as a test of the inner loop model. In real-world medical studies, where the low incidence of disease makes imbalanced data more common, and ML classifiers will provide a bias towards higher predictive accuracy for most classes, ML may face challenges when encountering class-imbalanced data [32]. The Synthetic Minority Oversampling Technique (SMOTE) is based on the feature space of the samples and increases the number of minority class samples in the dataset by interpolating them to synthesize the minority class samples to achieve sample balance with the majority class [33]. The SMOTE method allows the ML model to fully learn the features of the minority class samples, improve the generalization ability and accuracy of the model, and reduce overfitting [34]. The prevalence of gout in this study was 5.27% and there was a class imbalance in the data, thus the data needed to be SMOTE-processed. We used the SMOTE module (hyperparameters: sampling_strategy = ‘auto’, k_neighbors = 5, random_state = 42) of the imblearn library version 0.10.1 to handle the class imbalance of the data, and the ratio of labeled classes of 0 and 1 after processing was 50%: 50%. In addition, we calculated the similarity between the SMOTE-processed dataset and the original dataset using the Jaccard distance, and the results showed a high similarity. We normalized the feature data using the MinMaxScaler and processed the categorical variables using one-hot coding [35].

We used six classification ML algorithms including Light Gradient Boosting Machine (LGBM), Support Vector Machine (SVM), RF, Gradient Boosting Decision Tree (GBDT), Extreme Gradient Boosting (XGBoost), and Category Boosting (CatBoost) to predict gout associated with dietary fiber and TyG index. Since different ML algorithms have different properties when analyzing data, we trained and tested six different classification ML models. After comparing the AUC performance of each classification ML model, the LGBM model with the best AUC performance was selected as the optimal gout prediction model and the model was interpreted using SHAP.

### SHAP-based interpretable tools

SHAP combines game theory with local interpretation of machine learning models and generates a SHAP value for each model feature that identifies the value of the feature’s contribution to the outcome prediction, and a positive or negative SHAP value indicates that the feature positively or negatively affects the probability of the outcome prediction [36]. The importance of the model’s features and the contribution of each feature to the model’s output can be directly observed by plotting the SHAP summary plot, and the SHAP decision plot allows for observation of how the model makes decisions about the prediction of the outcome [37].

### Statistical analysis

Due to the complex survey design of NHANES, we used sample weights for analysis. Continuous variables were expressed as median (interquartile range) and categorical variables as counts (percentages) according to the distribution of variables. We grouped study participants by gout and used the Wilcoxon rank sum test for complex survey samples to compare differences between two groups for continuous variables and the Rao & Scott second-order corrected chi-square test for categorical variables. Use correlation heatmaps to analyze the correlation between variables. The area under the receiver operating characteristic (ROC) curve (AUC), accuracy, precision, recall, F1 score, brier score, and the area under the P-R curve (AP) of the six categorical ML models were summarized to evaluate the performance of the models. All statistical analyses were performed using R version 4.3.1 and Python version 3.11.5. R packages used included haven, tableone, gtsummary, survey, plyr, dplyr, tidyverse, arsenal, caret, ggcor, ggplot2 and missForest. ML analysis using Scikit-learn 1.2.2 library and imblearn library version 0.10.1 to handle class imbalance of data. A two-sided *P* < 0.05 was considered statistically significant.

## Results

### Baseline characteristics for study participants

A total of 12,645 participants (male 48.03%, female 51.97%) were included in this study, and Table 1 summarizes the demographic characteristics, lifestyle, medical history, laboratory parameters, and dietary data for participants in the gout and no-gout subgroups. The prevalence of gout among all participants was 5.27%, with a statistically significant difference between 70.46% males and 29.54% females (*P* < 0.001). The highest percentage of gout participants was 70.61% aged 50–79 years. Age, triglycerides (TG), UA, glycosylated hemoglobin (GHB), insulin, tobacco use, WC, BMI, hypertension, diabetes mellitus, TyG index, TyG-BMI index, and HOMA-IR were statistically significantly higher in the gout group than in the no-gout group (all *P* < 0.001). High-density lipoprotein (HDL), low-density lipoprotein (LDL), and dietary fiber were statistically significantly lower in the gout group than in the no-gout group (*P* < 0.001 for HDL, *P* = 0.004 for LDL, and *P* = 0.040 for dietary fiber). There was no statistical difference in alcohol consumption between the two groups (*P* = 0.400).

**Table 1:** Participants’ baseline characteristics weighted according to gout subgroups

				Gout group	
Characteristic	Overall	No, *N* = 11,978 (94.73%)		Yes, *N* = 667 (5.27%)	*P*-value
**Demographics**					
Age (years)	47.0 (33.0, 60.0)	47.0 (32.0, 60.0)		62.0 (50.0, 70.0)	< 0.001
Age group (years)					< 0.001
20–49	6,397 (50.59%)	6,289 (52.50%)		108 (16.19%)	
50–79	5,515 (43.61%)	5,044 (42.11%)		471 (70.61%)	
≥ 80	733 (5.80%)	645 (5.39%)		88 (13.20%)	
Gender					< 0.001
Female	6,572 (51.97%)	6,375 (53.22%)		197 (29.54%)	
Male	6,073 (48.03%)	5,603 (46.78%)		470 (70.46%)	
Race					0.400
Non-Hispanic White	3,038 (38.16%)	2,850 (37.85%)		188 (43.52%)	
Non-Hispanic Black	1,769 (22.22%)	1,660 (22.05%)		109 (25.23%)	
Other Race	1,244 (15.62%)	1,183 (15.71%)		61 (14.12%)	
Mexican American	1,069 (13.42%)	1,032 (13.70%)		37 (8.56%)	
Other Hispanic	842 (10.58%)	805 (10.69%)		37 (8.56%)	
Education attainment		11,966		667	0.500
Less than 9th grade	1,307 (10.35%)	1,232 (10.30%)		75 (11.24%)	
9-11th grade	1,785 (14.13%)	1,692 (14.14%)		93 (13.94%)	
High School graduate/GED or equivalent	2,866 (22.69%)	2,695 (22.52%)		171 (25.64%)	
Some college or AA degree	3,642 (28.83%)	3,448 (28.81%)		194 (29.09%)	
College graduate or above	3,033 (24.00%)	2,899 (24.23%)		134 (20.09%)	
PIR	2.88 (1.39, 5.00)	2.88 (1.39, 5.00)		2.91 (1.44, 4.91)	> 0.900
**Laboratory parameters**					
TG (mmol/L)	101.00 (70.00, 150.00)	100.00 (69.00, 148.00)		133.00 (87.00, 197.00)	< 0.001
UA (umol/L)	321.20 (267.70, 380.70)	321.20 (261.70, 374.70)		389.40 (321.20, 463.90)	< 0.001
HDL (mmol/L)	1.34 (1.11, 1.63)	1.34 (1.11, 1.66)		1.19 (1.01, 1.50)	< 0.001
LDL (mmol/L)	2.87 (2.30, 3.52)	2.87 (2.33, 3.54)		2.72 (2.02, 3.36)	0.004
VitD (nmol/L)	64.10 (46.41, 82.60)	64.10 (46.54, 82.40)		63.37 (43.29, 85.25)	0.900
SII	448.00 (325.15, 627.75)	447.69 (325.50, 626.21)		470.16 (315.73, 667.38)	0.200
GHB (%)	5.50 (5.20, 5.80)	5.40 (5.20, 5.70)		5.70 (5.40, 6.20)	< 0.001
TyG index	8.55 (8.14, 8.99)	8.54 (8.13, 8.97)		8.93 (8.46, 9.41)	< 0.001
TyG-BMI index	238.98 (200.48, 285.57)	237.65 (199.31, 282.69)		280.83 (233.27, 331.62)	< 0.001
Insulin (uU/mL)	9.90 (6.27, 16.07)	9.71 (6.21, 15.86)		14.24 (8.61, 23.26)	< 0.001
HOMA-IR	2.47 (1.48, 4.24)	2.42 (1.46, 4.15)		3.96 (2.29, 6.68)	< 0.001
**Lifestyles**					
Alcohol consumption (drinks/month)					0.400
1–5	5,016 (49.98%)	4,773 (50.05%)		243 (48.70%)	
5–10	784 (7.81%)	747 (7.83%)		37 (7.41%)	
More than 10	1,452 (14.47%)	1,362 (14.28%)		90 (18.04%)	
Non-drinker	2,784 (27.74%)	2,655 (27.84%)		129 (25.85%)	
Tobacco use					< 0.001
Never smoker	7,058 (55.85%)	6,776 (56.61%)		282 (42.28%)	
Former smoker	3,045 (24.10%)	2,772 (23.16%)		273 (40.93%)	
Current smoker	2,534 (20.05%)	2,422 (20.23%)		112 (16.79%)	
**Examination**					
WC (cm)	97.50 (87.00, 108.60)	97.00 (86.70, 107.90)		109.20 (99.00, 119.10)	< 0.001
BMI group (kg/^2^)					< 0.001
Underweight (< 18.5)	217 (1.72%)	212 (1.77%)		5 (0.75%)	
Normal (18.5 to < 25)	3,525 (27.88%)	3,423 (28.58%)		102 (15.29%)	
Overweight (25 to < 30)	4,176 (33.02%)	3,976 (33.19%)		200 (29.99%)	
Obese (30 or more)	4,727 (37.38%)	4,367 (36.46%)		360 (53.97%)	
**Medical history**					
Hypertension					< 0.001
No	7,520 (62.34%)	7,381 (64.53%)		139 (22.24%)	
Yes	4,543 (37.66%)	4,057 (35.47%)		486 (77.76%)	
Diabetes mellitus					< 0.001
No	10,767 (87.22%)	10,342 (88.34%)		425 (66.61%)	
Yes	1,578 (12.78%)	1,365 (11.66%)		213 (33.39%)	
**Dietary parameters**					
Total calories (kcal)	1,968 (1,509, 2,532)	1,969 (1,515, 2,531)		1,907 (1,406, 2,577)	0.300
Lycopene (mcg)	2,740.25 (772.50, 7,046.94)	2,743.30 (775.05, 7,095.11)		2,637.97 (672.44, 6,545.18)	0.500
Dietary fiber (gm)	15.45 (10.70, 21.65)	15.50 (10.75, 21.65)		14.20 (9.65, 21.21)	0.040
CDAI	-0.39 (-2.02, 1.71)	-0.38 (-2.01, 1.70)		-0.71 (-2.21, 1.78)	0.400

Continuous variables were expressed as median (interquartile range), categorical variables were expressed as counts (percentages).

PIR, family poverty income ratios; TyG index, triglyceride-glucose index; TyG-BMI index, TyG-body mass index; TG, triglycerides; UA, uric acid; HDL, high-density lipoprotein; LDL, low-density lipoprotein; VitD, 1, 25-hydroxyvitamin D; GHB, glycosylated hemoglobin; CDAI, composite dietary antioxidant index; HOMA-IR, homeostasis model assessment-IR; WC, waist circumference; BMI, body mass index; SII, systemic immune-inflammatory index.

### Correlation analysis between variables

**Fig. 2:**
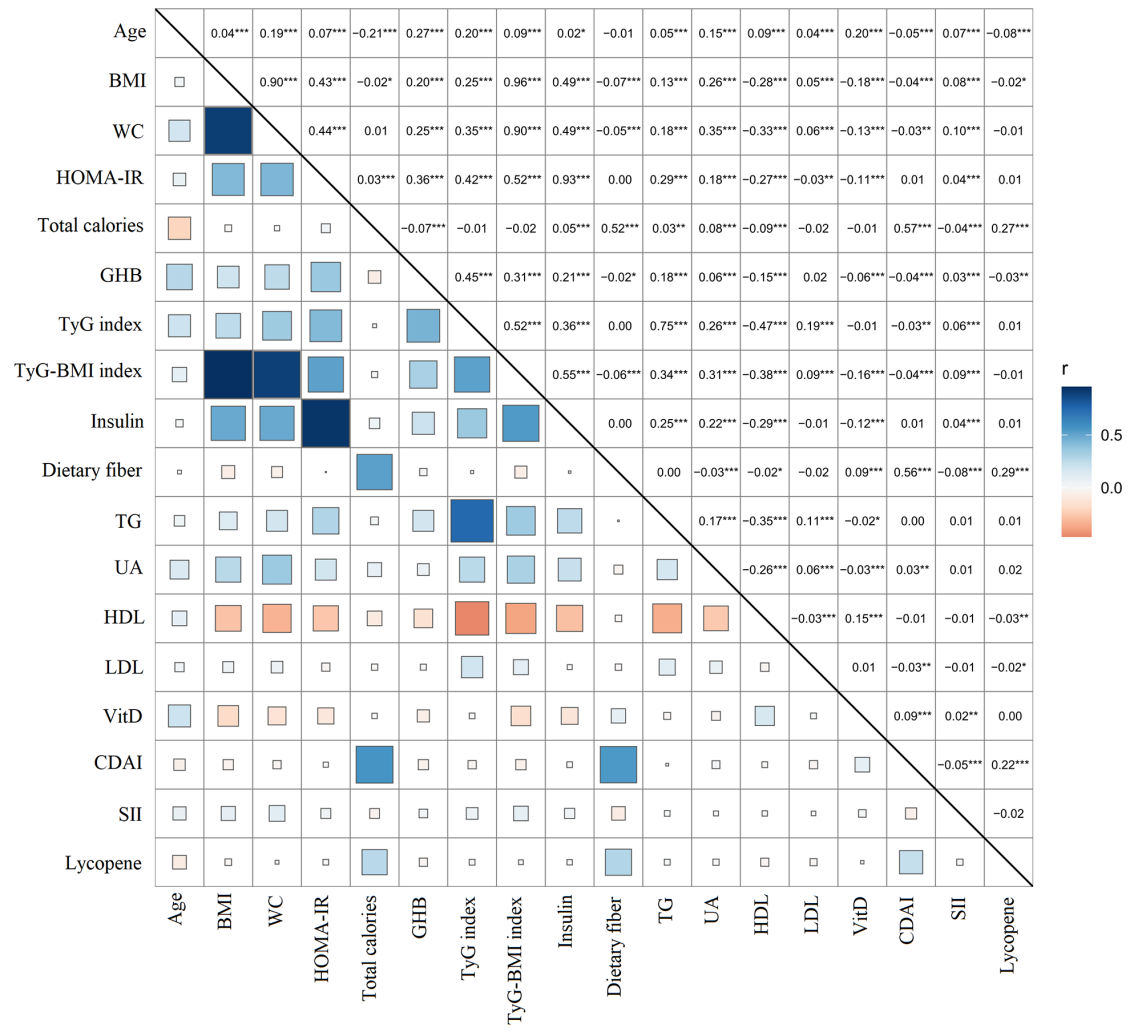
The correlation heat map of variables. The r represents Pearson’s correlation coefficient, * for *P* < 0.05, ** for *P* < 0.01, *** for *P* < 0.001, blue for positive correlation, and orange for negative correlation

Figure 2 demonstrates the correlation analysis between the variables. The results showed strong correlations between BMI with WC and TyG-BMI index, respectively, with Pearson’s correlation coefficients of 0.90 and 0.96 (*P* < 0.001). There were also strong correlations between WC and TyG-BMI index, HOMA-IR and Insulin, with correlation coefficients of 0.90 and 0.93 (*P* < 0.001), respectively. TyG index was positively correlated with age, UA, BMI, WC, HOMA-IR, and GHB with correlation coefficients of 0.20, 0.26, 0.25, 0.35, 0.42, and 0.45 (*P* < 0.001), respectively. There was a positive correlation between dietary fiber and total calories with a correlation coefficient of 0.52 (*P* < 0.001).

### ML model performance comparison and final confirmation

**Fig. 3:**
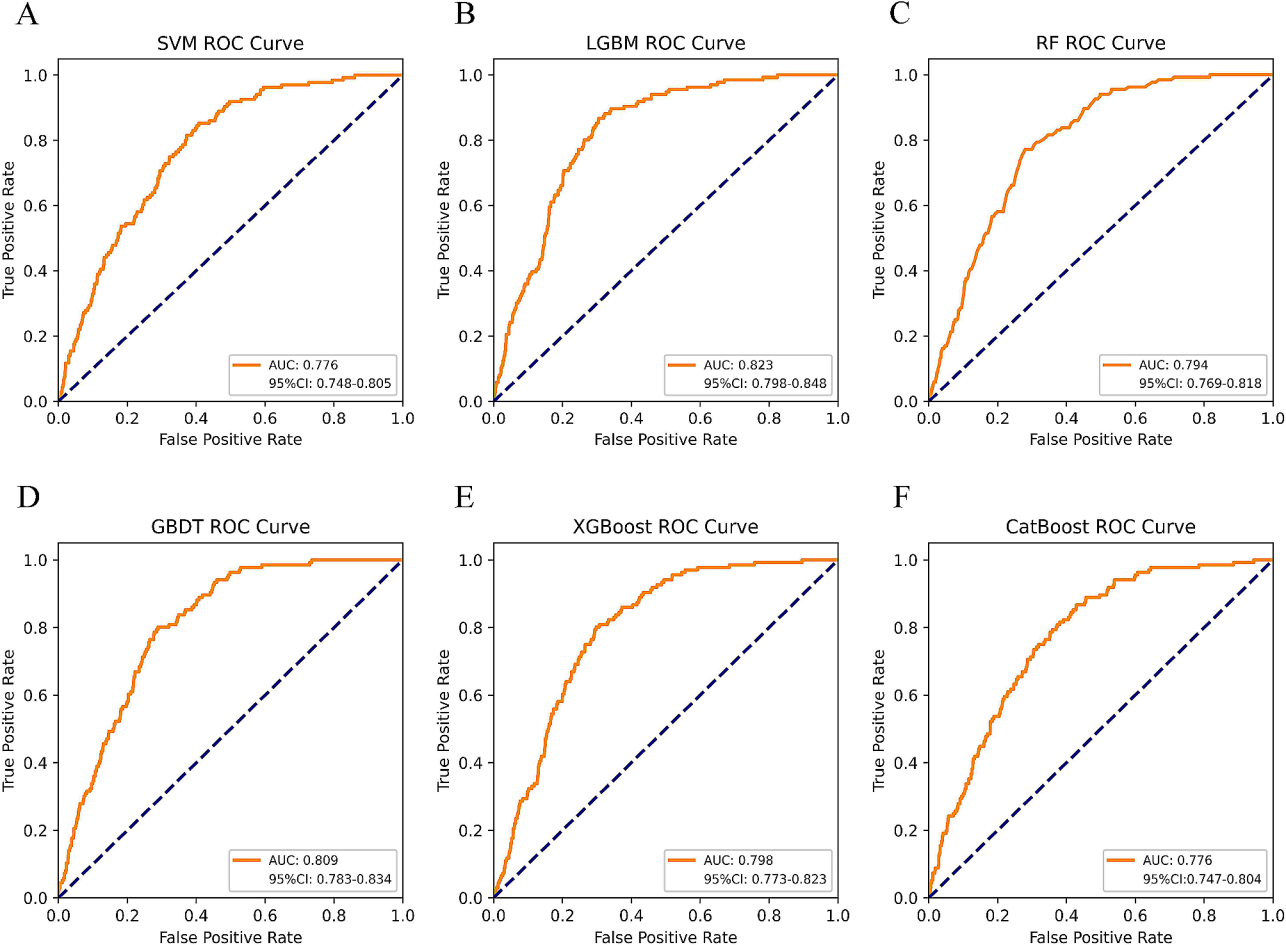
AUC comparison of six classification ML models

**Fig. 4:**
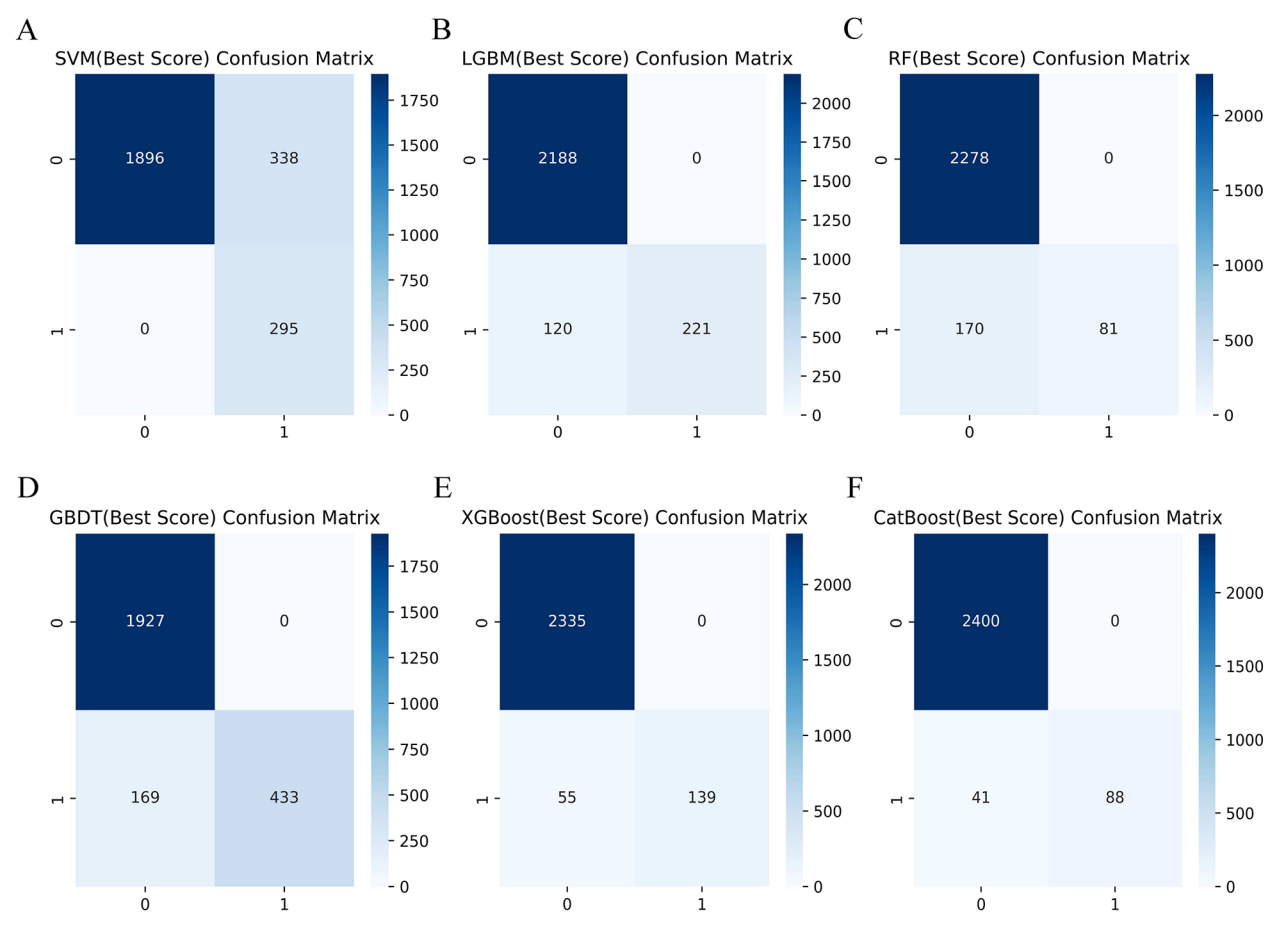
Confusion matrix comparison of six classification ML models

**Fig. 5:**
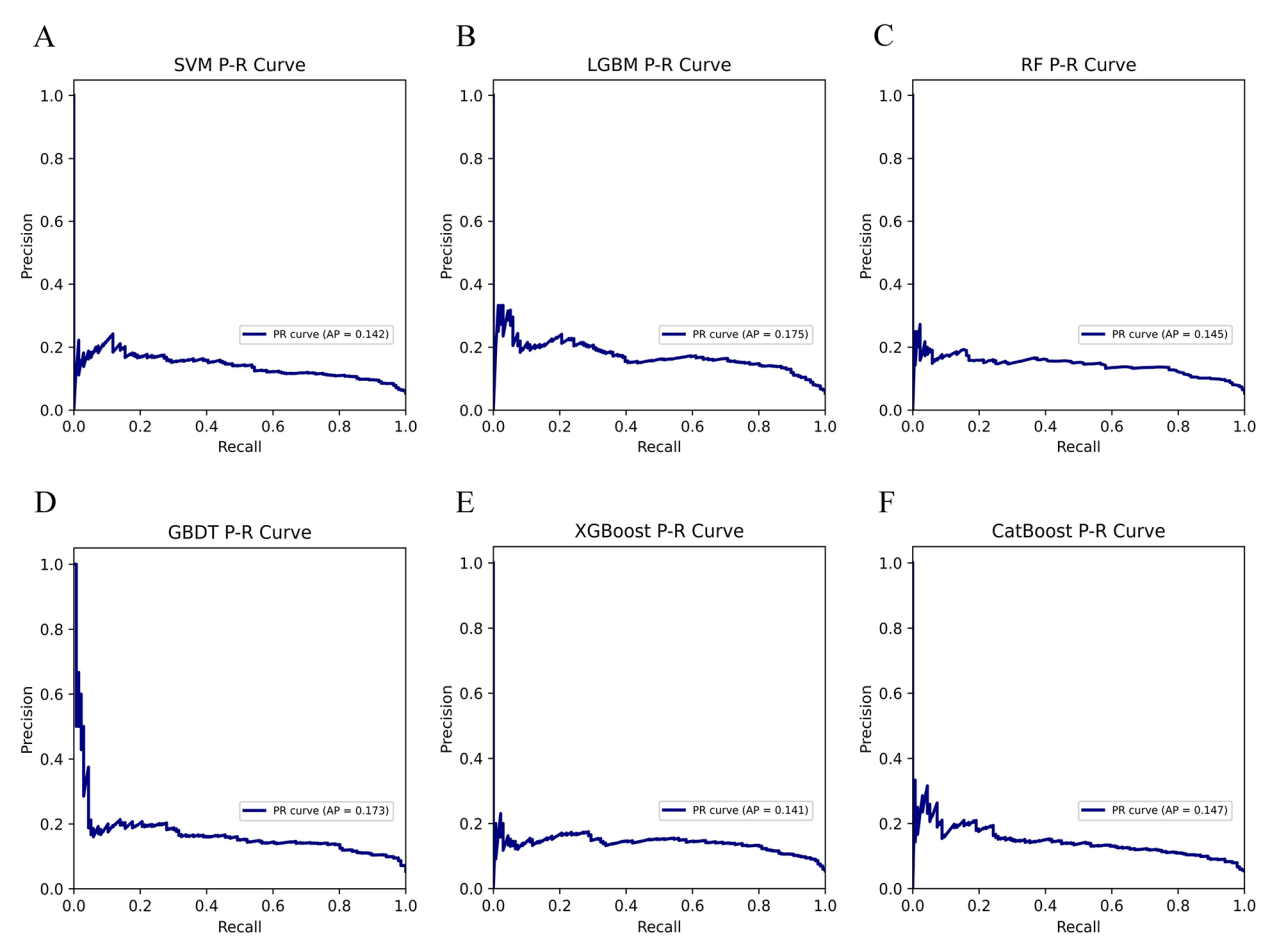
Comparison of P-R curves of six classification ML models

Table 2 summarizes the performance of SVM, LGBM, RF, GBDT, XGBoost, and CatBoost models in predicting gout. We used the Bootstrap method with 1000 resamples to estimate the 95% CI of the model AUC. Figure 3 shows that the LGBM classification ML model with an AUC of 0.823 (95% CI: 0.798–0.848) performed the best in predicting gout when compared to SVM, RF, GBDT, XGBoost and CatBoost models. A comprehensive analysis based on the discriminant features of the model showed that LGBM has high accuracy and robustness in predicting gout. The LGBM model has an accuracy of 95.3%, precision of 100%, recall of 64.8%, F1 score of 0.786, brier score of 0.077, and AP value of 0.175 (Figs. 4 and 5). The optimal hyperparameters for our finalized and saved LGBM model are learning_rate: 0.1, max_depth: -1, num_leaves: 31, min_data_in_leaf: 20, feature_fraction: 1, bagging_fraction: 1, bagging _freq: 0, max_bin: 255, n_estimators: 40.

**Table 2:** Performance comparison of six classification ML models

Characteristics	SVM	LGBM	RF	GBDT	XGBoost	CatBoost
AUC	0.776	0.823	0.794	0.809	0.798	0.776
AUC 95% CI	0.748–0.805	0.798–0.848	0.769–0.818	0.783–0.834	0.773–0.823	0.747–0.804
Accuracy	0.867	0.953	0.933	0.933	0.978	0.984
Precision	0.467	1.000	1.000	1.000	1.000	1.000
Recall	1.000	0.648	0.323	0.719	0.716	0.682
F1 score	0.636	0.786	0.488	0.837	0.835	0.811
Brier score	0.145	0.077	0.077	0.114	0.068	0.061
AP	0.142	0.175	0.145	0.173	0.141	0.147

SVM, support vector machine; LGBM, light gradient boosting machine; RF, random forest; GBDT, gradient boosting decision tree; XGBoost, extreme gradient boosting; CatBoost, category boosting; AUC, the area under the ROC; AP, the area under the P-R curve

### ML model features importance visualization

**Fig. 6:**
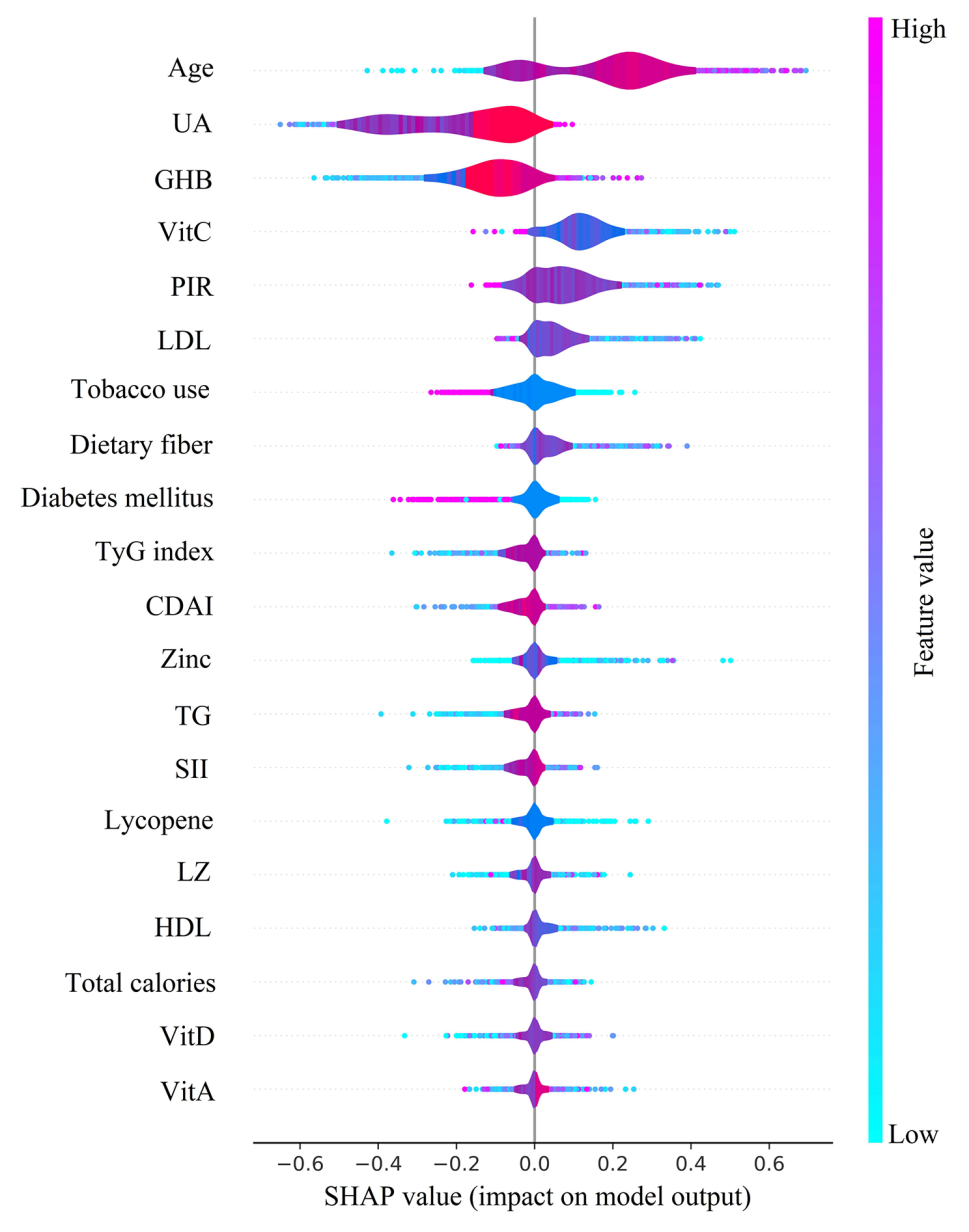
SHAP summary plot of LGBM model predicting gout

We used SHAP summary plots to show the effect of the top 20 features across the dataset on predicting gout after fitting the LGBM model (Fig. 6). The importance of SHAP plot features showed that age was the most important feature of the LGBM model for predicting gout. The SHAP values indicated that age (SHAP value: 0.356), VitC (SHAP value: 0.355), LDL (SHAP value: 0.338), HDL (SHAP value: 0.285), dietary fiber (SHAP value: 0.174), SII (SHAP value: 0.106), BMI (SHAP value: 0.081), CDAI (SHAP value: 0.074), Se (SHAP value: 0.072), and TyG index (SHAP value: 0.068) all positively influenced the predictions of the model. While SHAP values showed GHB (SHAP value: -0.179), VitA (SHAP value: -0.110), LZ (SHAP value: -0.106), Zinc (SHAP value: -0.089), UA (SHAP value: -0.088), insulin (SHAP value: -0.069), total calories (SHAP value: -0.049), lycopene (SHAP value: -0.043), TG (SHAP value: -0.034), and VitD (SHAP value: -0.019) negatively influenced the predictions of the model. In addition, the figure shows that older age, higher UA, lower dietary fiber intake, and higher TyG index all lead to an increased risk of gout.

### Interpretability of individual decision-making processes

**Fig. 7:**
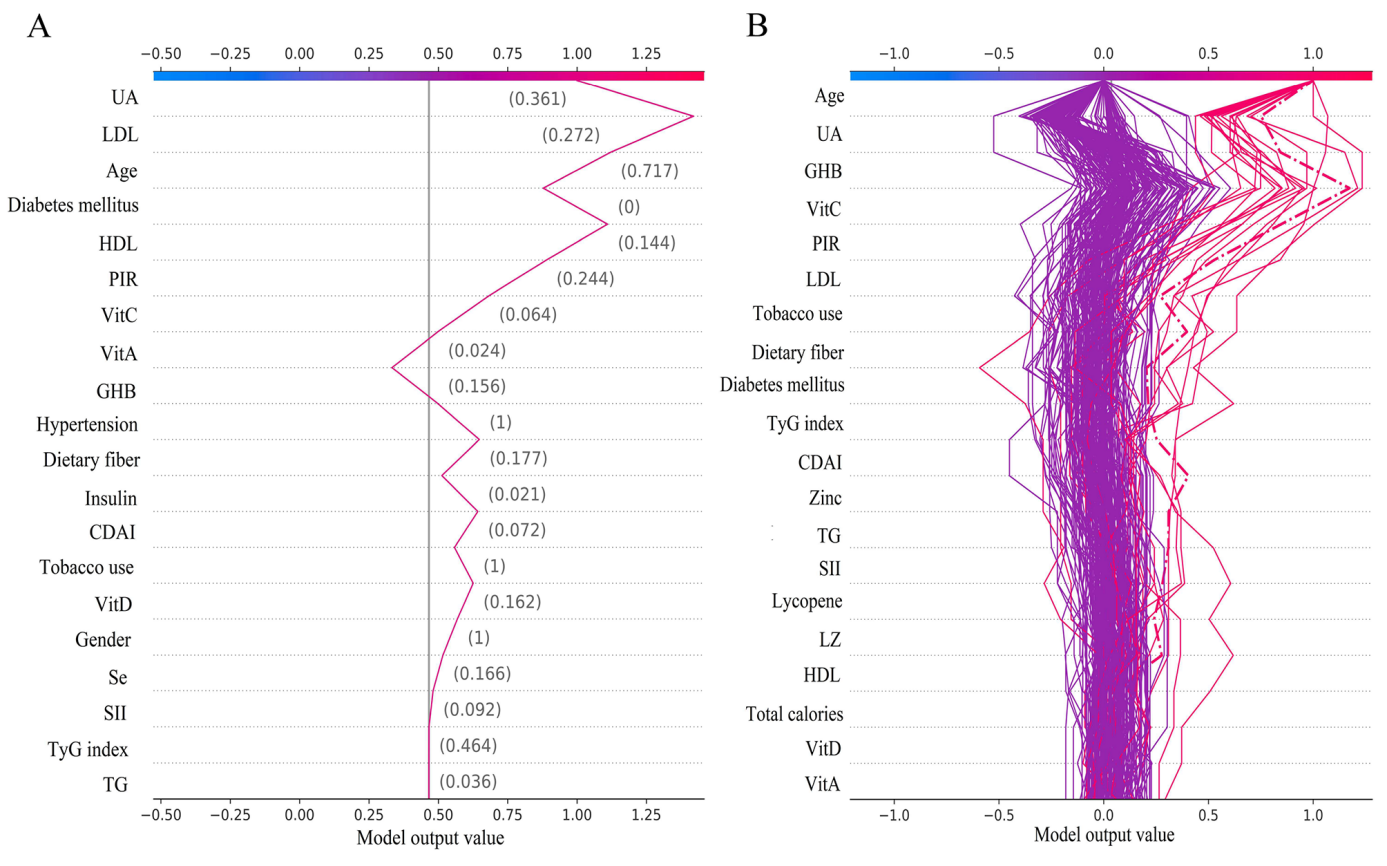
The LGBM model SHAP decision plot. (**A**) Shows how individuals make decisions to predict gout from the LGBM model. (**B**) How the top 200 individuals in the LGBM model make decisions to predict gout

The decision plot shows how individuals determine their decision to predict gout from the complex LGBM categorization model. The gray vertical line in the middle of the decision plot is the base value for the categorical LGBM model, and the red line indicates a positive prediction, marking whether or not each feature moved the output value above or below the average prediction, and marking the value of each feature (Fig. 7A). Starting at the bottom of the decision plot, the prediction lines show how the SHAP values accumulate from the base value to the final gout prediction result of the model at the top of the plot, ultimately boiling down to 0 for a negative prediction and 1 for a positive prediction. The decision plot shows the top 200 individual predictions, with the purple line being the negative prediction and the red line being the positive prediction, highlighting the first individual prediction (Fig. 7B).

## Discussion

We trained and tested six classification ML models for predicting gout using the NHANES dataset from 2005 to 2018. Based on the performance of the model on unknown data, the LGBM model outperforms SVM, RF, GBDT, XGBoost, and CatBoost. The SHAP method is used to interpret the LGBM model, which provides global and local interpretability of the model. This will help clinicians better understand the model’s decision-making process and trust the model more. The AUC of the LGBM model was 0.823 (95% CI: 0.798–0.848), reflecting excellent classification model efficiency and stability with an accuracy of 95.3%. We used SHAP global summary plots to demonstrate the importance of each feature in the LGBM model and how much each feature contributes to the model’s prediction of gout. Furthermore, we found that age was the most important characteristic among the variables used to predict gout in the LGBM model, followed by UA. The decision plots provide a clear and intuitive demonstration of how individuals make decisions in the categorical LGBM model.

Zou F et al. studied the relationship between the composition of fruits and UA suggesting that the viscosity and bulk of dietary fiber hinder the absorption of purines and fructose in the digestive tract and reduce the rate of small intestinal reabsorption and that dietary fiber may also facilitate defecation and promote the excretion of UA [38]. Guo Y et al. found that inulin, a non-digestible dietary fiber, can significantly ameliorate hyperuricemia in mice by studying the effect of inulin on mice with hyperuricemia due to knockout of the UA oxidase gene [39]. In a multicenter retrospective study, Wu Z et al. pointed out that the TyG index, a novel and reliable surrogate for IR, and hyperuricemia are both important metabolic risk factors, that are closely related to each other and contribute to each other through multiple mechanisms [40]. Zhao Q et al. demonstrated a nonlinear relationship between serum UA and TyG index in children with short stature, which remained nonlinear after gender stratification [41]. Hyperuricemia is an important influence in causing gout [42], thus supporting our findings.

This study differs from the prediction of gout and correlation analysis using traditional analysis. Studies have shown that ML consistently outperforms traditional analytics in predicting disease and that the use of more sophisticated integrated classification algorithms can be effective in improving the accuracy and robustness of predicting disease [43]. In recent years, AI has been accumulating great success in medical research and applications at an alarming rate, AI encompasses a large number of different technologies, and ML is a sub-field of AI that provides separate tools to enable most of the techniques of AI [44]. In addition, ML has many distinct advantages. First, it can learn from large amounts of information, discover hidden patterns and laws, and excel at handling complex problems and big data. Second, it can provide more powerful predictive capabilities than traditional methods. Third, it can automate many tasks and improve computational efficiency. Fourth, it can give data-driven probabilistic predictions. However, ML also faces challenges. First, it requires a large amount of sample data for computation; Second, there is a potential risk of overfitting; and third, the accuracy of the model is highly dependent on the reliability of the data to reflect clinical reality [45].

The performance of an ML algorithm depends on many factors, the most important of which is often not the algorithm itself but reliable data and representative features [46]. The quality and quantity of data determines the upper limit of the performance of an ML algorithm, and the selection of appropriate features determines the ability of an ML algorithm to represent and generalize the data. Once we have acquired reliable data and suitable features, increasing the interpretability of the predictive model becomes the main problem we need to face. However, the inner workings of traditional ML are often ambiguous, with the nature of an “algorithmic black box” and the inability to see the process of conclusions, which greatly restricts the confidence of healthcare practitioners in their use, and the transparency and interpretability of ML models have become a hot research direction for researchers [47]. Therefore, it is important that we finalize an interpretable LGBM model associated with dietary fiber and TyG index for the prediction of gout to provide individualized preventive and therapeutic plans for populations at potential risk of gout.

The LGBM classification model we trained for predicting gout using NHANES data has many strengths and limitations. First, we use SHAP to make the behavior and predictions of the LGBM model easier to interpret and to make the decision or prediction process easier to understand in complex models. Second, to avoid the substantial labor and economic costs of data collection and ill-designed data surveys, demographic, laboratory, dietary interview, and disease self-report questionnaire data from NHANES were utilized to screen for relevant 27 study features. Third, NHANES is nationally representative and has high-quality data. The predictive models developed in conjunction with ML have national utility and are superior to predictive models developed from localized census data and models developed using traditional analytical methods. Fourth, our study was a cross-sectional investigation and could not confirm causality, as well as the fact that the diagnosis of gout was based on participants’ self-reports, which could be potentially confused by memory.

## Conclusion

The LGBM model associated with dietary fiber and TyG index showed high accuracy, efficiency, and robustness in predicting gout, and may provide individualized prevention and treatment strategies for people with potential gout risk. Increasing dietary fiber intake and lowering the TyG index may reduce the potential risk of gout. We will further validate and generalize the medical applicability of this predictive model by expanding and updating the data with ongoing follow-up analysis of selected features to improve the interpretability of the categorical LGBM model.

### Electronic supplementary material

Below is the link to the electronic supplementary material.

Supplementary Material 1. The model candidate variables.

## Data Availability

The datasets used and/or analyzed during the current study are available from the corresponding author upon reasonable request.

## Abbreviations

UA: Uric acid
IR: Insulin resistance
TyG: Index Triglyceride-glucose index
ML: Machine learning
AI: Artificial intelligence
NHANES: National Health and Nutrition Examination Survey
SHAP: SHapley Additive exPlanations
NCHS: National Center for Health Statistics
BMI: Body mass index
HOMA-IR: Homeostasis model assessment-IR
SII: Systemic immune-inflammatory index
VitA: Vitamin A
VitC: Vitamin C
VitD: 1, 25-hydroxyvitamin D
VitE: Vitamin E
Se: Selenium
LZ: Lutein + zeaxanthin
CDAI: Composite dietary antioxidant index
RF: Random Forest
SMOTE: Synthetic minority oversampling technique
LGBM: Light Gradient Boosting Machine
SVM: Support vector machine
GBDT: Gradient Boosting Decision Tree
XGBoost: Extreme Gradient Boosting
CatBoost: Category Boosting
ROC: Receiver operating characteristic
AUC: Area under the ROC
AP: Area under the P-R curve
TG: Triglycerides
GHB: Glycosylated hemoglobin
HDL: High-density lipoprotein
LDL: Low-density lipoprotein
PIR: Family poverty income ratios
