# Supplementary material for: Interpretable machine learning framework to predict gout associated with dietary fiber and triglyceride-glucose index
Source: Nutr Metab (Lond). 2024 May 14;21:25. doi: 10.1186/s12986-024-00802-2 (PMC11092237; doi:10.1186/s12986-024-00802-2)
Supplement: Supplementary file 1 — Supplementary Material 1. The model candidate variables. [file 12986_2024_802_MOESM1_ESM.docx]

The initial variables include gender, age, PIR, alcohol consumption, tobacco use, BMI, waist circumference, HOMA-IR, total calories, GHB, TyG index, TyG-BMI index, insulin, dietary fiber, TG, UA, diabetes mellitus, HDL, LDL, VitD, CDAI, SII, hypertension, lycopene, VitA, VitC, VitE, Zinc, Se, LZ, gout, height, weight, apolipoprotein B, and high-sensitivity C-reactive protein.
